# Hearing loss and cognitive function among older adults in 18 high-income countries: Moderating and Mediating factors

**DOI:** 10.21203/rs.3.rs-7371554/v1

**Published:** 2025-10-27

**Authors:** Xu Zong, Huaiyue Wang, Guoqi Xiao, Ye Zhang

**Affiliations:** University of Helsinki; Shanghai Normal University; Beijing Normal University; Renmin University of China

**Keywords:** Hearing loss, Cognitive health, Machine learning, cross-national analysis

## Abstract

**Background:**

Hearing loss is a major, potentially modifiable risk factor for cognitive impairment, but its moderating and mediating factors remain underexplored in cross-national settings.

**Methods:**

We used the data from a cross-national survey, the Survey of Health, Ageing and Retirement in Europe (SHARE). 38,506 participants aged 65 and above across 17 European countries and Israel from SHARE wave 6 were included. The study applied the Double Machine Learning (DML) approach to evaluate the association of hearing aid use with cognitive function in older adults, and to test demographic moderators and psychosocial mediators.

**Results:**

Hearing loss was significantly associated with poorer cognitive performance in temporal orientation (coefficient = −0.061; 95% CI: [−0.076, −0.045]), memory (–0.448; 95% CI: −0.520, −0.375]), numeracy (–0.061; 95% CI: [−0.092, −0.030]), and verbal fluency (–0.379; 95% CI: [−0.529, −0.229]). The associations varied by gender, education and age group, such as stronger associations with verbal fluency of women, and memory of higher educated individuals. Mediation analyses indicated that the negative associations of hearing loss with cognitive function may be partially explained by increased feelings of isolation, loneliness and depression.

**Conclusion:**

This study highlights the cognitive burden of hearing loss in later life and underscores the importance of early detection and intervention. Addressing psychosocial consequences of hearing impairment may help mitigate its impact on cognitive health. Further longitudinal research is needed to validate these findings and clarify causal pathways.

## 1. Introduction

Aging populations are increasing globally[1]. During the demographic transition, it is critical to understand the determinants of cognitive function since cognitive decline is prevalent among older adults [2, 3] and currently, there is no cure for severe cognitive impairment [4]. For instance, the rate of cognitive impairment in Europe is projected to be between 5.1% and 24.5% [5], with many replying on long-term care. Numerous studies have explored both protective and risky determinants of cognitive function among older adults. Protective factors identified include social activity, and engagement in artistic, craft [6]. In contrast, risky factors encompass depressive symptoms, onset of hypertension, vascular diseases, and chronic conditions [7].

The Lancet Commission report pointed that, hearing impairment is one of the nine risk factors for dementia that are potentially modifiable[1]. Poor hearing performance was associated with increased cerebrospinal fluid tau protein levels, with analysis indicating that brain structure and CSF tau protein partially explained its link to cognitive decline [8]. Hearing loss also demands greater cognitive resources for auditory processing [9], which can detract from other cognitive function like working memory. Furthermore, the association between hearing loss and cognitive function may not be uniform across populations. Prior research has shown that gender, age, and education levels may moderate the association between hearing loss and cognitive outcomes [10, 11], suggesting that sociodemographic differences may shape the vulnerability or resilience to the association between hearing loss and cognitive function. Additionally, evidence has demonstrated the association between hearing loss and cognitive decline through several mechanistic pathways. The hearing loss can lead to verbal communication difficulties, thereby reducing social engagements and increase the risk of depression and social isolation [12–15], which are key risks of cognitive impairment. Therefore, we propose the following hypotheses:

Hypothesis 1: Hearing loss is association with the decrease of cognitive function among older adults.

Hypothesis 2: The association between hearing loss and cognitive function may vary across gender, age and education levels.

Hypothesis 3: The association between hearing loss and cognitive function may be mediated by feeling depressed, feeling lonely, and feeling isolated.

Previous research has primarily focused on small sample sizes, often from a single country or region [16–19]. Additionally, conventional regression models are susceptible to unobserved or unmeasured confounding factors. These limitations may compromise both the statistical power and the generalizability of previous findings. This study aims to use cross-nationally representative data and a machine learning approach to examine the association between hearing loss and cognitive function among older individuals in 17 European countries and Israel. We also aim to identify the heterogeneity across subgroups and potential mechanisms in this association.

## 2. Data and method

### 2.1 Data

#### 2.1.1 Data Description

This study drew data from the Survey of Health, Aging, and Retirement in Europe (SHARE), which encompasses participants aged 50 and above in 28 European counties and Israel. The dataset includes various information about demographics, family structure, health status and functioning, and others [20]. The ethical review for SHARE waves 1 to 4 was approved by the Ethics Committee of the University of Mannheim, while wave 4 and subsequent waves obtained ethical approval from the Ethics Council of Max Plank Society. To evaluate the association of hearing loss with cognitive function, we used the data from SHARE wave 6 fielded in 2015, which included 68, 085 participants. We selected wave 6 because the variables of cognitive function have a high rate of missing values in waves 7 and 8, and earlier waves (waves 1–5) covered fewer countries. It is necessary to include hearing aid use as the control variable since evidence shows hearing aid use may moderate the impact of hearing loss on cognitive function [21]. After excluding individuals under the age of 65 and those with missing data on key variables, the final analytic sample comprised 38,506 participants.

#### 2.1.2 Measurements

Following the measurement of a previous study [22], this study measures cognitive function by four domains including temporary orientation, memory, numeracy, and verbal fluency. Temporary orientation was measured by the orientation to date, month, year and day of the week, scored from 0 to 4, with higher scores indicating better oriented. Memory was assessed by summing the immediate and delayed word recall scores, ranging from 0 to 20. Numeracy was measured by asking the participants to subtract 7 in five consecutive trials, with correct answer scored from 0 to 5. Verbal fluency was measured by asking participants to name as many animals as possible within one minute for testing the speed and ease of verbal production, with scores ranging from 0 to 100.

In SHARE Wave 6, participants were asked to rate their hearing (while using a hearing aid, if applicable) as excellent, very good, good, fair, or poor. In this study, we constructed a binary variable for hearing loss, classifying responses of ‘fair’ or ‘poor’ as indicating hearing loss, and ‘excellent’, ‘very good’, or ‘good’ as indicating no hearing loss.

Cognitive function in late life is associated with a range of sociopsychological and biological determinants [23–26]. Therefore, the study included 22 sociopsychological and biological determinants from six domains as confounders, including demographics (gender, age, marital status, education levels, living in a rural or urban area, living in Eastern, Western, Southern Europe, or Israel), physical, mental health status (ever had high blood pressure, ever had diabetes, ever had cancer, ever had lung disease, ever had heart problems, ever had stroke, body mass index), health behaviors (drinking, smoking), economic situation (having public pensions, household total income), social network (weekly contacting with children), childhood circumstances (mother education, father education, mathematics performance, language performance).

To examine the potential mechanisms, we included several mediators, including isolation (feeling isolated from others), loneliness (3-item loneliness summary mean score), and depression (EURO-D score).

### 2.2 Method

We employed Double machine learning (DML), an approach developed by Chernozhukov et al. (2018), recently used to evaluate the intervention effects, such as the Special Supplemental Nutrition Program for Women, Infants, and Children [28–30]. Compared to traditional statistical models, DML offer significant advantages, including the ability to handle non-parametric functions and incorporate a large number of confounders [31]. Therefore, DML’s flexibility in estimating non-parametric relationships between confounders and outcomes enables robust analysis.

In this study, we used DML to examine the association of hearing loss with cognitive function among older adults. There are two key considerations why DML was chosen as the approach for this study: (i) Cognitive function is influenced by a range of interrelated factors, such as demographic, health status, and health behavioral variables, which often show complex and non-linear interactions. Traditional statistical methods such as linear regression models rely on strong parametric assumptions that may oversimplify these relationships, potentially resulting in biased or inaccurate results. (ii) DML has the ability to incorporate high-dimensional confounders and effectively capture complex, non-linear interactions without the risk of overfitting. By using DML, this study provides more reliable and unbiased estimates of the association between hearing loss and cognitive function, effectively controlling for potential biases introduced by confounders. The analysis incorporates 22 sociopsychological and biological covariates to ensure robust and accurate findings.


(1) CFi=θH0Li+g(Xi)+Uit,E(Ui|Xi,HLi)=0

(2) $$HL_{i}=m\left(X_{i}\right)+V_{i},\;E\left(V_{i}|X_{i}\right)=0$$

As Eq. (1) and Eq. (2) shows, we constructed a partially linear double machine learning model, where *i* means participants, CF means cognitive function of older adults, *HL* indicates the occurrence of hearing loss, *X*_*i*_ indicates confounder, *g* (*X*_*i*_) and *m* (*X*_*i*_) indicate the specific form identified by machine learning, *U*_*i*_ and *V*_*i*_ means error term. Lasso algorithm was used for regression predictions of both the dependent variable (cognitive function) and the treatment variable (hearing loss), using 5-fold cross-validation to enhance generalization. Residuals were analyzed using linear regression to estimate coefficients, and this process was repeated across folds to produce the final average estimated coefficients. So, this approach combines the predictive ability of machine learnings with accurate estimation capability of linear regression, reducing biases in assessing the association of hearing loss with cognitive function among older adults. We made the analysis in Stata (version 18.0), with all results presented a 95% confidence interval.

## 3. Results

### 3.1 Descriptive statistics

Main demographic characteristics of participants in the SHARE wave survey 6 are summarized in the Supplementary materials. The sample consists of 38,506 individuals with a mean age of 66.58 years; 55% were female, and 66% reported being married or having a partner. Among all participants, 73.93% reported no hearing loss during the survey period, while 26.07% reported experiencing hearing loss. Participants with hearing loss had significantly lower scores across four domains of cognitive function (p < 0.001) and were more likely to be older, less educated, unmarried or without a partner, male, and distributed in Southern or Eastern Europe compared to those without hearing loss (p < 0.001).

### 3.2 Association of hearing loss with cognitive function

**Figure 1:**
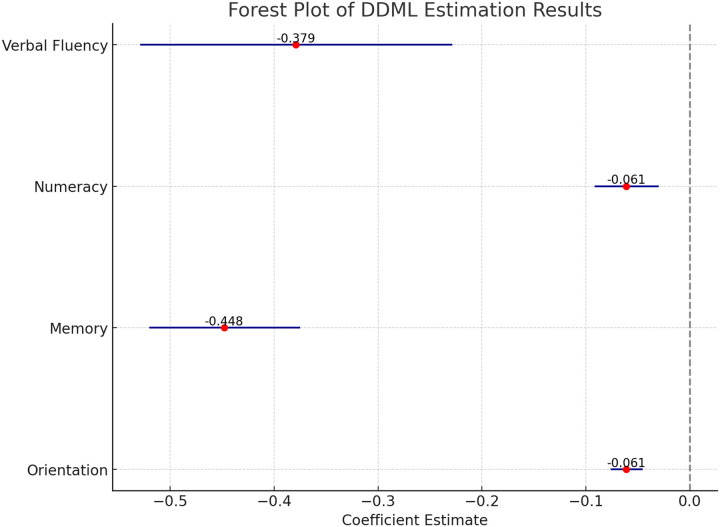
Estimates of the association between hearing loss and cognitive function Note: All control variables were included. The red dots represent the coefficients, while the blue horizontal lines indicate the 95% confidence intervals.

The findings indicate that hearing loss is significantly linked to declines across all four cognitive domains: verbal fluency, numeracy, memory, and temporary orientation (see Fig. 1). Specifically, compared to participants without hearing loss, those with hearing loss scored lower by 0.379 points in verbal fluency (95% CI: −0.529, −0.229), 0.061 points in numeracy (95% CI: −0.092, −0.030), 0.448 points in memory (95% CI: −0.520, −0.375), and 0.061 points in temporary orientation (95% CI: −0.076, −0.045).

### 3.3 Mediating analysis

Table 1 presents the results of the mediation analysis examining the indirect effects of hearing loss on cognitive function through potential mediators. Specially, increased isolation, loneliness and depression significantly mediated the associations between hearing loss and four domains of cognitive function.

### 3.4 Heterogeneity analysis

The study explores the heterogeneous association between hearing loss and cognitive function across gender, age and education levels.

#### Gender heterogeneity

**Figure 2:**
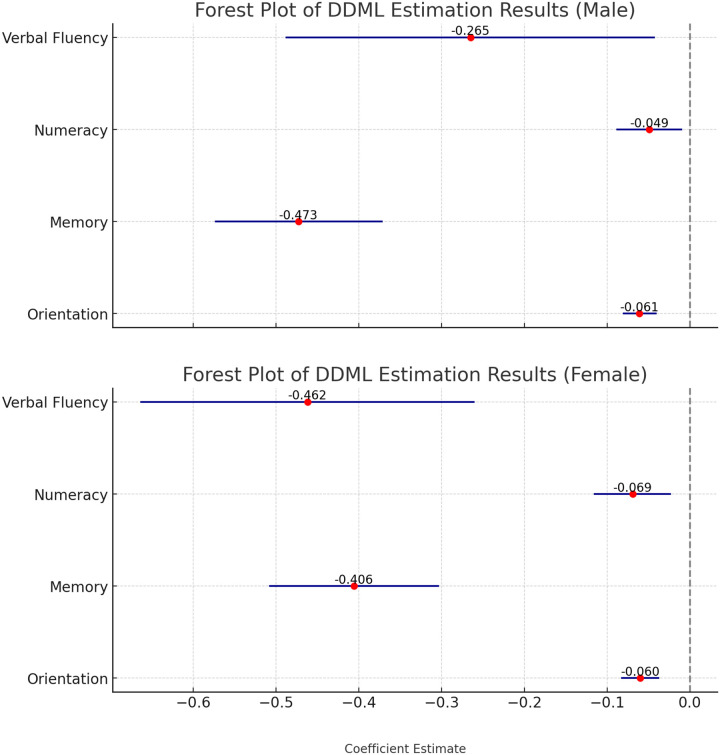
Estimates of the association between hearing loss and cognitive function by gender

Older adults exhibit significant gender differences in cognitive function [32, 33], which may influence the relationship between hearing loss and cognitive decline. To account for this, the study conducted heterogenous analyses across gender (see Fig. 2). The estimated coefficients for both genders are significantly negative across all four cognitive domains. However, the magnitude of the effects varies: hearing loss has a greater impact on verbal fluency, numeracy and orientation in women, while men experience a larger decline in memory compared to women.

#### Education heterogeneity

**Figure 3:**
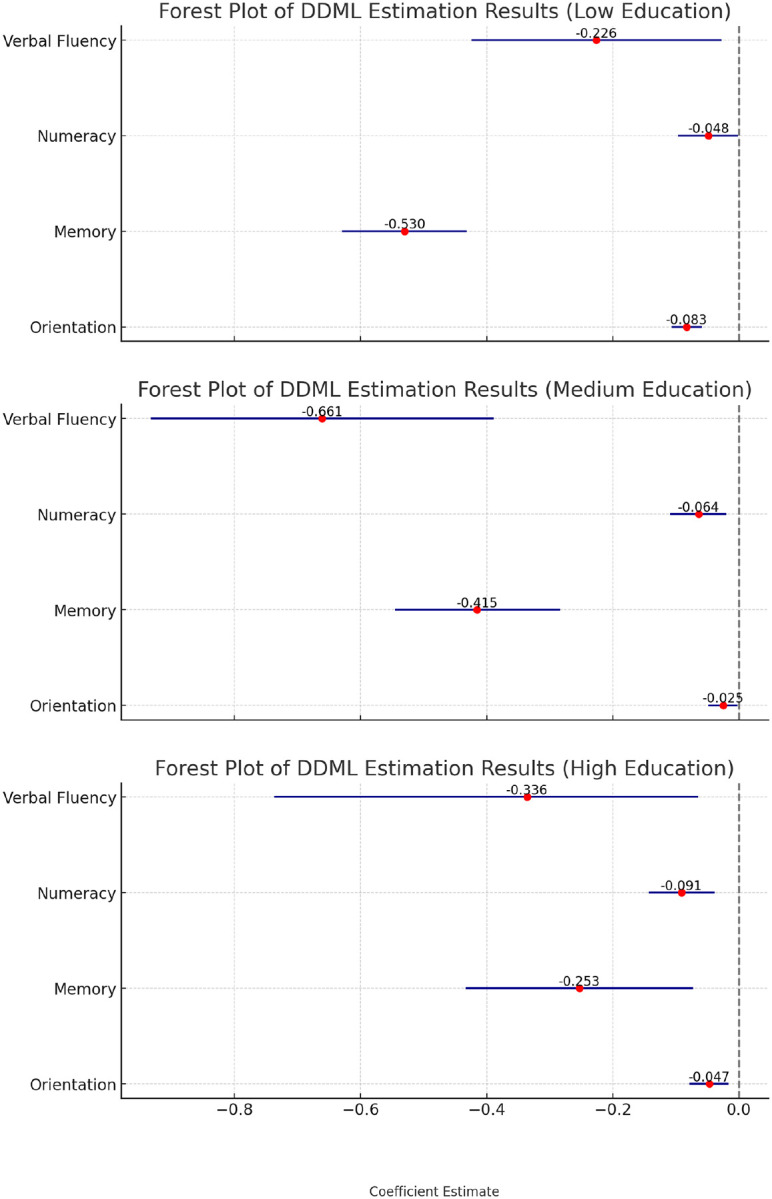
Estimates of the association between hearing loss and cognitive function by education levels Note: Low education indicates less than upper secondary education, medium education indicates upper secondary and vocational training, and high education indicate tertiary education.

Education was closely linked to cognitive function [34, 35]. To examine its role in the relationship between hearing loss and cognitive decline, this study examined the heterogeneity across different education levels (see Fig. 3). The negative association between hearing loss and numeracy was more pronounced among older adults with higher education levels (tertiary education), whereas the association on memory was stronger among those with lower education levels (less than upper secondary education).

#### Age heterogeneity

**Figure 4:**
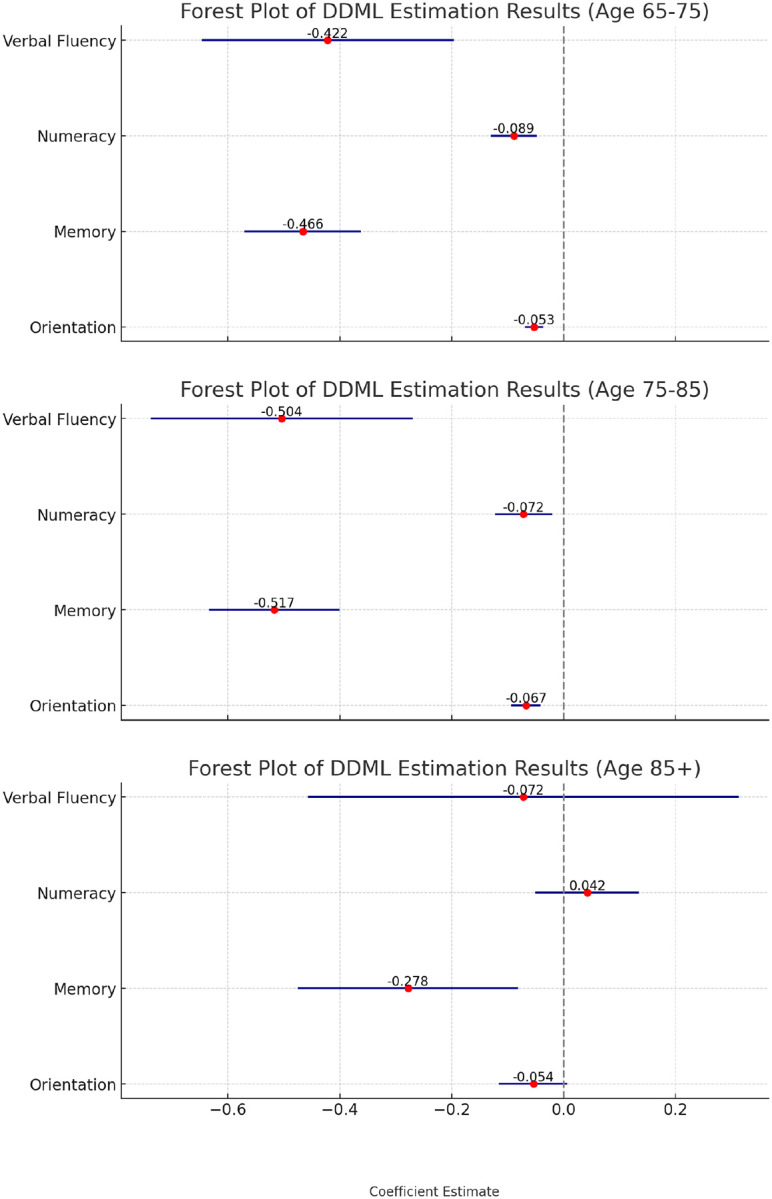
Estimates of the association between hearing loss and cognitive function by age

We examined the heterogenous associations between hearing loss and cognitive function across age groups (see Fig. 4). Notably, for adults 85 and older, the association between hearing loss and cognitive function was not statistically significant in three of the four domains. Additionally, the association between hearing loss and numeracy appears stronger among the younger age groups (younger than 75 years old).

### 3.5 Robust analysis

We conducted the robust analysis from three aspects. First, we replaced the hearing loss variable from a binary indicator (having or not having hearing loss) to a continuous scale ranging from 1 (excellent hearing) to 5 (poor hearing). Second, we adjusted the k-fold cross-validation from 5 to 3 and 7. Third, we modified the machine learning algorithm, replacing the previously used lasso with random forest, gradient boosting, and neural networks. As shown in the Supplementary materials, whether adjusting the variable specification, modifying the k-fold cross-validation, or altering the machine learning algorithms, the findings remain stable: hearing loss was associated with poorer cognitive function.

## 4. Discussion

In the context of the growing aging population and demographic transitions, this study is one of the first to investigate the association of hearing loss with cognitive function using cross-national data and DML approach. The findings showed that hearing loss was significantly associated with lower cognitive function across four domains: verbal fluency, numeracy, memory, and temporal orientation. Moreover, analysis revealed that the association was mediated through three factors: feelings of isolation, loneliness, and depression. The study further revealed that this negative association varied by gender, education and age.

Consistent with previous studies, this research demonstrated a significantly association between hearing loss and lower cognitive function [36, 37]. Several hypotheses had been proposed to explain the potential mechanisms underlying this relationship [38]. The sensory deprivation hypothesis suggested that long-term hearing loss reduced auditory input to the brain, leading to structural and functional changes that negatively affected cognitive processes. The information degradation hypothesis posited that reduced quality of auditory information due to hearing loss required greater cognitive effort for processing, thereby diverting cognitive resources away from other tasks. In addition, the common cause hypothesis argued that both hearing and cognitive decline may arise from shared neurodegenerative processes, such as brain atrophy or impaired neural connectivity.

This study also found that isolation, loneliness, and depression mediated the relationship between hearing loss and cognitive function. This aligned with previous research showing that hearing loss can disrupt communication, which in turn reduced social engagement and increases emotional distress like loneliness and depression—factors known to contribute to cognitive decline [13, 14, 39, 40]. Moreover, evidence regarding the mediating roles of depression and loneliness remained mixed. For example, studies based on American and Chinese samples had not found these pathways to be statistically significant [13, 41]. Such inconsistencies may stem from differences in sample characteristics and model designs [42]. This study used a double machine learning approach on a larger and more diverse European sample, with more extensive covariate adjustment.

Furthermore, the study suggested that the association between hearing loss and cognitive function varied across gender, education, and age subgroups. The findings showed that hearing loss had a stronger negative impact on women in verbal fluency, which is consistent with prior research [10]. One possible explanation was that women engaged more widespread brain regions during language processing tasks [43]. When hearing declined, the associated structural and functional changes in the brain may have a greater impact on their language-related cognitive performance. In terms of education heterogeneity, previous studies had suggested that higher education may buffer the cognitive impact of hearing loss by higher cognitive reserve [44]. However, the findings of this study revealed domainspecific differences: hearing loss had a stronger negative impact on numeracy among individuals with higher education, while its impact on memory was more pronounced among those with lower education. This may be because individuals with higher education generally performed at a higher baseline in numeracy tasks [45], so even modest impairment due to hearing loss may result in more noticeable declines. As for age heterogeneity, this study found that the negative association between hearing loss and cognitive function was more pronounced among adults aged 65 to 85, but became statistically insignificant in most cognitive domains among those aged 85 and above. This contrasted with some prior research suggesting that the association remained consistent across age groups [46]. The discrepancy may stem from methodological differences, as this study uses double machine learning rather than mixed-effects models and a broader set of control variables.

Based on the findings, this study proposed the following policy recommendations: First, given the significant association between hearing loss and cognitive decline, hearing health should be integrated into the broader framework of health management for older adults. Public health initiatives should aim to raise awareness about hearing impairment and its potential cognitive consequences, thereby promoting early detection and timely intervention. Second, the impact of hearing loss on cognitive function varied across gender, educational, and age groups. Accordingly, policies and interventions should adopt a differentiated approach, offering more tailored and targeted cognitive support services for high-risk subpopulations. Third, the analysis showed that the cognitive effects of hearing loss are partially mediated through isolation, depression, and loneliness. Therefore, interventions should not only address sensory health but also consider broader psychological factors.

The strengths of this study lied in four aspects. First, this study used data of 38,506 samples from 18 high-income countries. This large-scale, multi-national data allowed us to evaluate the association of hearing loss with cognitive function in a wider cultural context and improved the generalizability of the research results. Second, this study used DML approach, which can handle high-dimensional data and effectively control a large set of confounders, thereby more robustly evaluating the relationship between hearing loss and cognitive function. Third, this study distinguished four specific domains of cognitive function rather than using a global score, providing a more nuanced analysis. Finally, it explored the mechanisms and heterogeneity across gender, education, and age, providing further insight into population-specific effects of hearing loss.

The study also had several limitations. Hearing loss and some confounders (e.g., economic status, physical and mental health conditions) were self-reported in our study, which had the potential risk of misreporting. Another limitation was that this study was a cross-sectional design and therefore it was not possible to establish causal associations between hearing loss and cognitive function.

## 5. Conclusion

The findings of this study indicated that hearing loss was significantly associated with lower cognitive function across multiple domains. This association varied by gender, education, and age groups, and was mediated by isolation, loneliness, and depression. These findings underscored the importance of integrating hearing health into comprehensive aging and cognitive health strategies, and of designing tailored interventions that address the specific needs of different demographic groups.

## Supplementary Files

This is a list of supplementary files associated with this preprint. Click to download.

- Supplementarymaterials.docx

## Figures and Tables

**Table 1:** Estimated indirect effects of potential mediators linking hearing loss to cognitive function.

Mediators	Isolation	Loneliness	Depression
**Temporal orientation**	−0.007*** (−0.010, −0.005)	−0.008*** (−0.011, −0.005)	−0.030*** (−0.034, −0.025)
**Memory**	−0.048*** (−0.062, −0.034)	−0.049*** (−0.063, −0.034)	−0.158*** (−0.180, −0.136)
**Numeracy**	−0.018*** (−0.023, −0.013)	−0.021*** (−0.026, −0.015)	−0.046*** (−0.054, −0.037)
**Verbal fluency**	−0.103*** (−0.132, −0.074)	−0.099*** (−0.130, −0.069)	−0.323*** (−0.369, −0.277)

Note:

*** < 0.001,

** < 0.01,

* < 0.05,

95% confidence interval in parentheses.

## Data Availability

The dataset analyzed in this study is publicly available at the following website: www.share-eric.eu.
